# A simple and effective technique for laparoscopic gastrorrhaphy: modified Graham’s patch with barbed suture

**DOI:** 10.1186/s12893-023-02192-3

**Published:** 2023-09-27

**Authors:** Ta-Chun Chou, Chun-Hui Lee, Ruey-Shyang Soong, Yi-Chan Chen

**Affiliations:** 1 Department of Surgery, Keelung Branch, Chang Gung Memorial Hospital, No. 222, Maijin Rd., Anle Dist, 204201 Keelung City, Taiwan; 2 Division of Transplantation, Department of Surgery, Taipei Municipal Wan-Fang Hospital, Taipei Medical University, Taipei, Taiwan; 3 College of Medicine, Medical University, Taipei, Taiwan

**Keywords:** Perforated peptic ulcer, Peritonitis, Laparoscopic, Barbed suture, Modified Graham’s patch

## Abstract

**Introduction:**

Peptic ulcers are caused by unbalanced acid production, and proton pump inhibitors (PPIs) in recent decades have helped to treat peptic ulcers effectively. Meanwhile, the incidence of perforated peptic ulcer (PPU) persists and has a high mortality rate if there is no adequate management. Primary closure with a modified Graham’s patch was well performed in early detected PPU with a small size < 2 cm. A laparoscopic approach for PPU was prescribed for decades with proven feasibility and safety. We introduced an effective technique combined with barbed suture and modified Graham’s patch, which can significantly reduce the surgical time without significantly increasing morbidity and mortality compared with traditional interrupted suture.

**Patients and method:**

We retrospectively collected data from January 2014 to December 2020 in Keelung Change Gung Memorial Hospital, and a total of 154 patients receiving laparoscopic repair of PPU were included. There were 59 patients in the V-loc group (V group) and 95 patients in the laparoscopic primary repair group (P group).

**Results:**

The V group had a significantly shorter operation time than the P group (96.93 ± 22.14 min vs. 123.97 ± 42.14, *P* < 0.001). Ten patients suffered from morbidity greater than the Clavien‒Dindo classification 4 (5 from V group, and 5 from P group). Three patients with leakage were reported. Two patients were in the V group, and one patient was in the P group (p = 0.432).

**Conclusion:**

Laparoscopic repair with barbed suture and modified Graham’s patch provides a simple and effective technique in the management of acute abdomen. This technique can be easily performed by experienced surgeons and trainees in minimally invasive surgery without affecting patient safety.

## Introduction

Peptic ulcer disease (PUD) is caused by unbalanced acid production, which destroys the mucosal barrier. The stomach pyloric area and duodenum first portion are the most common sites of perforation. Medical treatments, including antibiotics for *Helicobacter pylori* (H. pylori) eradication and proton pump inhibitor (PPI) use, have decreased the incidence of PUD [1]. However, the incidence of perforated peptic ulcer (PPU) persists at approximately 2–10% for patients with PUD and still plays an important role in health care worldwide. Patients suffering from PPU had a high mortality rate of up to 20–25% in previous reports [2, 3] owing to delayed diagnosis-related sepsis and complications after the operation, such as pneumonia or multiorgan failure. Intensive care and advanced interventions have improved in recent decades, but high mortality makes PPU a challenge for all surgeons.

The management of patients with PPU includes aggressive resuscitation, nasogastric tube decompression, intravenous antibiotics, PPI use, and surgical source control [4]. The surgical approach for patients with PPU has been advocated from distal hemigastrectomy with vagotomy for acid control in the past to simple closure with or without omental patches/plugs recently, and these procedures have also moved to the era of minimally invasive surgery in recent decades [5]. Modified Graham’s patch repair for PPU was reassessed as an effective procedure to give excellent outcome in wound healing and lower the comorbidity. Laparoscopic repair of the PPU was first conducted in 1990, and several randomized clinical trials favored the clinical outcome of laparoscopic repair or at least noninferior to the traditional approach with less pain, less bowel manipulation and shorter hospital stay [6]. However, several reviews revealed that the operative time was longer in laparoscopic repair owing to the relatively difficult techniques of intracorporeal suture for young surgeons without enough experience compared to the open group [7]. A longer operation time would cause extra time for anesthesia and pneumoperitoneum, which may lead to more postoperative complications, such as lung atelectasis complicated by pneumonia and cardiovascular events, especially in elderly patients. The procedure for emergent surgery should be simple and effective to provide better outcomes with fewer complications.

We introduce a simple but novel technique for the simple closure of the PPU with a pedicled omental patch, which can dramatically shorten the operation time, especially for young surgeons without much experience in laparoscopic surgery. This technique was prescribed with V-loc by performing a continuous suture of the perforation combined with an omental patch that could be anchored to the perforation stably with the V-loc(;(Medtronic/Covidien, New Haven, CT)). This design of the suture material promises the stability of the suture compared with the intracorporeal knotting technique, which may cause knot loosening by inexperienced hands. Under this simple procedure, we can also train senior residents in the technique of advanced laparoscopic operation.

## Materials and methods

### Patients

**Fig. 1:**
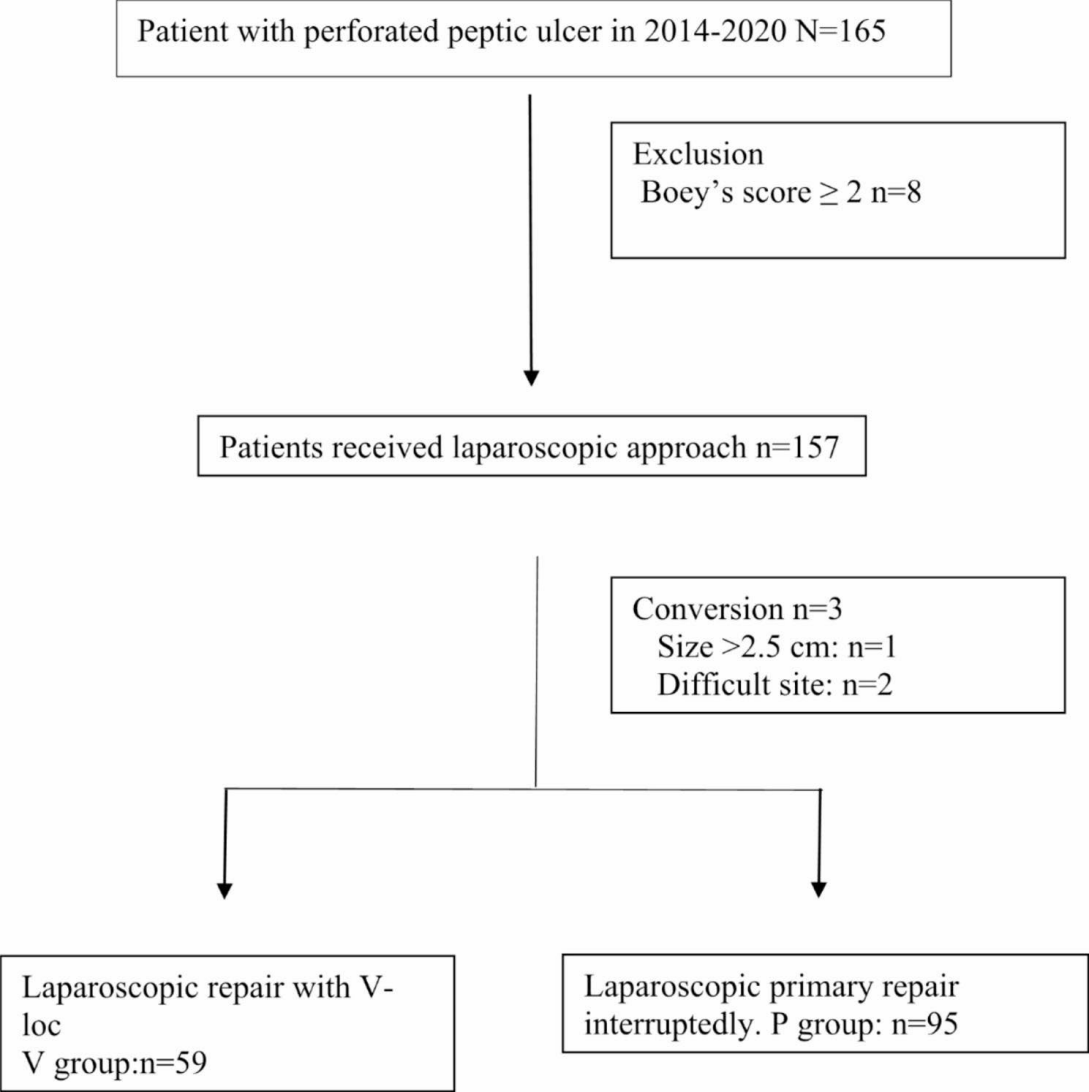
Algorithm for patient selection in laparoscopic treatment for perforated peptic ulcer

We retrospectively reviewed patients who received laparoscopic repair for perforated peptic ulcers at Keelung CGMH between January 2014 and December 2020. We used Boey’s score for pre-operative patient selection and only patients with Boey’s sore ≤ 1 were suggested to receive laparoscopic approach and otherwise open surgery was suggested. The flow chart of patient selection was presented in Fig. 1. The patients were divided into 2 groups: laparoscopic V-loc repair (V group) and laparoscopic primary repair (P group).The recruited patients’ clinical characteristics (age, sex, body mass index, Charlson Comorbidity Index (CCI), peptic ulcer perforation (PULP) score, Mannheim Peritonitis Index (MPI), Boey’s score, American Society of Anesthesiology (ASA) class, etc.), perioperative variables (operative time, blood loss, perforation site/size), and postoperative outcomes (postoperative pain scale, timing of NG removal and feeding, length of hospital stay) were retrieved from the prospectively collected database. Patients who did not have detailed preoperative/intraoperative clinical records or who did not have regular postoperative follow-up were excluded from our study. All the data collected are under the approval of the Institutional Review Boards (IRB) of Chang Gung Memorial Hospital (CGMH) (IRB NO: 202300742B0).

### Operative techniques

**Fig. 2:**
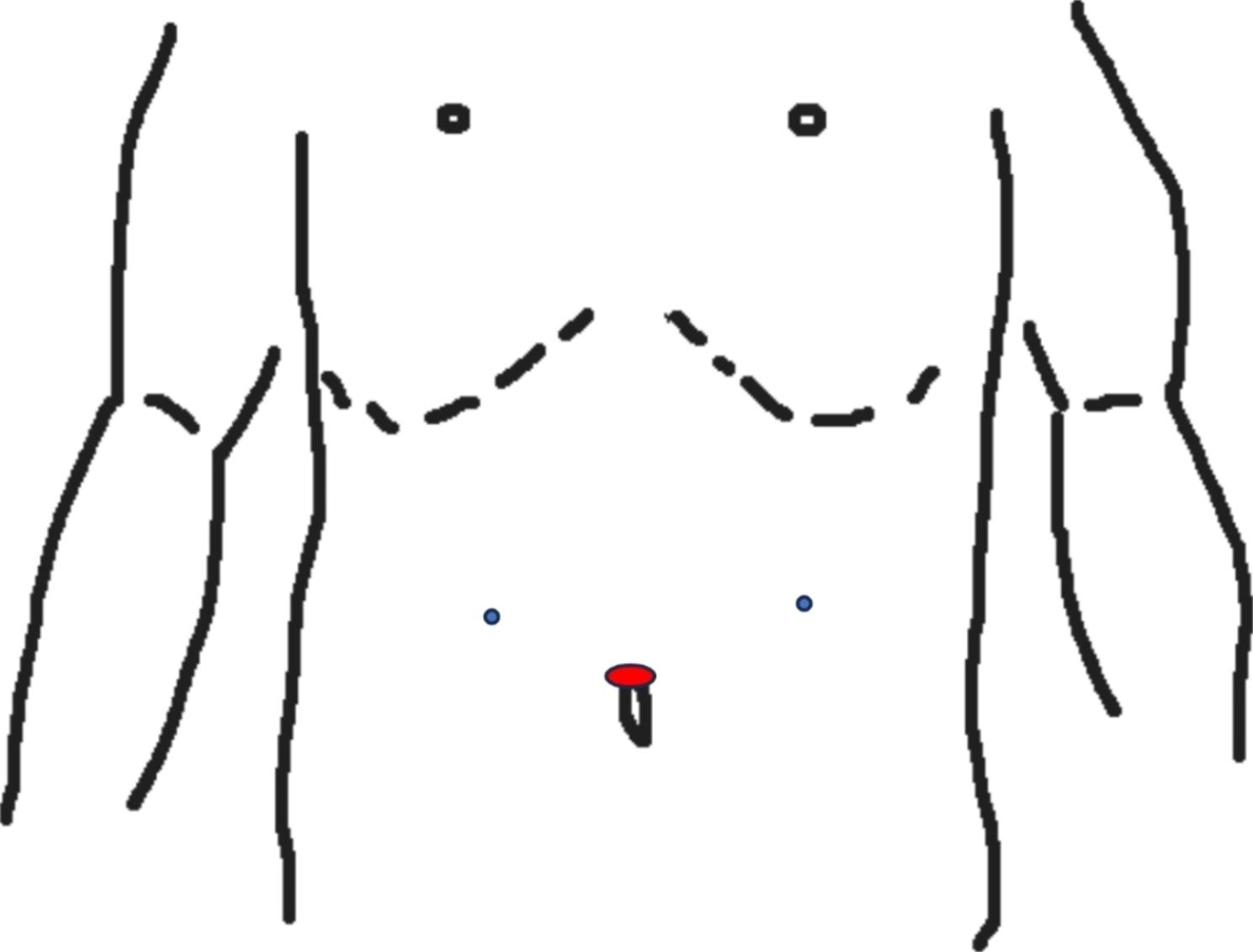
Sites of trocar insertion : The supraumbilical 12 mm port is the camera port and the other small 5 mm ports are working ports

**Fig. 3:**
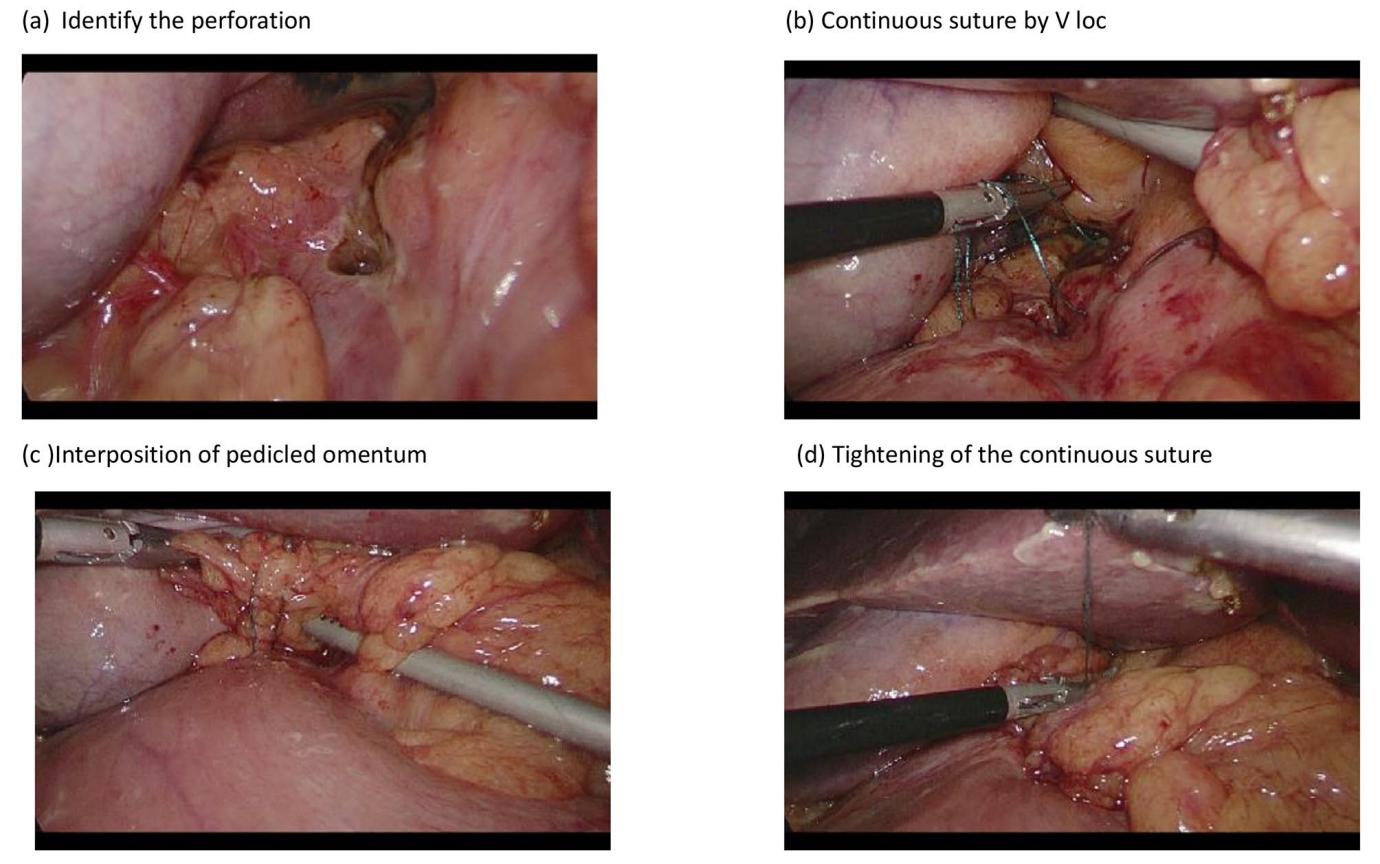
Step by step demonstration of surgical procedures

The patient was placed in the supine position without opening the legs, and the surgeon stood on the left side of the patient. The sites of trocar insertion are shown in Fig. 2. We used three trocars for exploration and suturing. The first 12 mm trocar was inserted with the Hasson mini-laparotomy technique to prevent bowel injury, and pneumoperitoneum was performed to 12–15 mmHg according to the patients’ response to the pneumoperitoneum. The other two 5 mm trocars were inserted on the bilateral side of the first trocar to create a working space. The patient was then rotated to the reversed Trendelenburg position with the right side upward to prevent the occupation of the liver on the working space during our repair. The surgeons used a 10-mm 30-degree videoscope to perform this operation. After exploration of the whole abdomen, the dirty ascites was drained via the suction-irrigation tube to decrease the infectious burden. The perforation was identified under the videoscope, and the perforation size was evaluated. Resection wounds should be considered instead of repair if the perforation is larger than 2 cm in diameter. After confirmation of the perforation, one piece of the pedicled omentum was taken by the unipolar scissor, and the V-loc was introduced into the peritoneal cavity via the 12 mm trocar. After the first cross suture of the perforation, the posterior small hole of the V-loc was used to replace the intracorporeal tie, and then another suture was performed without tightening. The piece of pedicled omentum was then inserted under the suture line after two cross sutures to cover the perforation, and then the suture line was tightened. One Jackson-Pratt (JP) drain was inserted after copious irrigation and confirmation of the leakage for further monitoring. The whole procedure was presented with a step-by-step image in Fig. 3.

### Statistics

The statistical analysis was performed with IBM SPSS Statistics 21 (IBM Corporation, Software Group, Somers, NY, USA). Fisher’s exact test or Pearson’s χ2 test was used to analyze categorical data. Student’s t test was used to analyze continuous variables. Statistical significance was defined as *P* values < 0.05 in two-sided tests.

## Results

There were 59 patients in the V group and 95 patients in the P group. The procedures were well tolerated in both groups. The patients’ demographic data are summarized in Table 1. There were no significant differences in terms of patient age, sex, Charlson Comorbidity score (CCI), Peptic Ulcer Perforation (PULP) score, Mannheim Peritonitis Index (MPI), preoperative white blood count (WBC), hemoglobin, C reactive protein (CRP) level, or body mass index (BMI) between the two groups.

**Table 1:** Demographic data of the patients

N = 154	Laparoscopic V	Laparoscopic P	P value	
Patients, n	59	95		
Gender (%)				
Male	42	69		
Female	17(29%)	26(27.4%)	*0.846*	
Mean Age (years,SD)	62.2 ± 17.6	58.46 ± 16.68	*0.185*	
Charlson Cormobidity Index	3.85 ± 2.37	3.41 ± 2.56	*0.29*	
Mannheim Peritonitis Index (MPI) Score	21.61 ± 4.57	21.06 ± 3.66	*0.41*	
Peptic Ulcer Perforation (PULP) score	3.78 ± 2.82	2.97 ± 2.68	*0.08*	
ASA classification				
0–2	33	65		
3–4	26	30	*0.117*	
Boey’s score				
0–1	59	92		
2		3	*0.168*	
Previous upper abdominal operations (%)	2(3.4%)	1(1%)	*0.308*	
Pre-OP				
WBC (1000/uL)	12.09 ± 5.26	11.77 ± 5.33	*0.72*	
Hb (g/dL, SD)	13.2 ± 2.97	13.9 ± 2.25	*0.11*	
Cr	1.13 ± 0.89	1.04 ± 0.65	*0.49*	
ALT (g/dL, SD)	24.72 ± 19.42	22.93 ± 16.07	*0.54*	
Bilirubin	0.53 ± 0.28	0.65 ± 0.47	*0.08*	
Na	136.99 ± 3.96	137.83 ± 3.16	*0.15*	
K	4.01 ± 0.57	4.36 ± 3.41	*0.43*	
CRP (mg/L, SD)	29.66 ± 61.09	19.29 ± 68.15	*0.34*	
Albumin	3.50 ± 0.57	3.59 ± 0.60	*0.47*	
BMI (mg/dL, SD)	22.4 ± 4.4	22.4 ± 3.75	*0.981*	

ASA: American society of anesthesia Pre-OP: pre-operation, WBC: white blood cell, Hb: hemoglobin ,Cr:creatinine, ALT: alanine aminotransferase, CRP: C-reactive protein, BMI: body mass index

For surgical variables, the V group had a significantly shorter operation time than the P group (96.93 ± 22.14 min vs. 123.97 ± 42.14, *P* < 0.001). Perforation size showed no difference (7.03 ± 6.51 mm vs. 8.64 ± 12.14 mm, *P* = 0.35). All patients received omentum patch enhancement. No serious intraoperative complications were recorded in either group (Table 2). The short-term postoperative outcomes following laparoscopic correction of PPU are shown in Table 3. The pain scale on postoperative day 1 (POD1), timing of nasogastric tube removal and length of hospital stay showed no significant difference between the two groups (*P* = 0.86, *P* = 0.17, *P* = 0.169). The implementation of enteral feeding was earlier in the V group (POD 4.64 ± 3 vs. 5.83 ± 2.61, *P* = 0.012), but the recovery time of the full diet showed no differences (POD 6.75 ± 3.59 vs. 7.72 ± 3.07, *P* = 0.082). Postoperative complications were recorded with the Clavien‒Dindo classification (CDC). Ten patients suffered from mortality more than CDC class 4 (5 from the V group and 5 from the P group). Three suture leakages were reported. Two patients were in the V group, and one patient was in the P group (p = 0.432). One of the V-loc leakage patients only showed minor leakage, recovered well after adequate drainage and was discharged under stable conditions. Another V-loc leakage patient refused further surgical correction, requested only conservative treatment, and expired due to sepsis due to profound shock. The leakage patient in the P group had received further exploratory laparotomy but still expired due to progressive sepsis.

**Table 2:** Operative data

N = 154	Laparoscopic V	Laparoscopic P	P value
Patients, n	59	95	
Mean operative time(mins, SD) Ranges	96.93 ± 22.14	123.97 ± 42.14	*< 0.001*
Estimate blood loss, ml Range	7.03 ± 6.51	8.64 ± 12.14	*0.35*
Perforation site			
Duodenum	12(20%)	21(22%)	
Prepyloric/antrum	47(80%)	74(78%)	*0.39*
Perforation size	0.75 ± 0.45	0.86 ± 0.39	*0.1*

**Table 3:** Post-operative outcome

N = 154	Laparoscopic V	Laparoscopic P	P value
Patients	59	95	
Early post-OP outcome			
POD1 pain scale NRS	2.42 ± 0.71	2.44 ± 0.79	0.86
Remove NG tube	3 ± 2.71	3.88 ± 4.23	0.17
Postoperative feeding	4.64 ± 3	5.83 ± 2.61	0.012
Diet as tolerate.	6.75 ± 3.59	7.72 ± 3.07	0.082
Discharge	8.93 ± 4.69	10.29 ± 6.36	0.169
Clavien-Dindo classification	9(15.3%)	10(10.5%)	0.515
2–3	4	5	
4–5	5	5	
Non-surgical related	4(6.8%)	5(5.3%)	*0.485*
Lung complications	1	4	
Renal complications	0	1	
Recurrent ulcer bleeding	1	0	
Other(cancer)	2	0	
Surgical related	5(8.5%)	5(5.3%)	*0.432*
Surgical site infection	1	2	
Postoperative ileus	2	2	
Suture leakage	2	1	

## Discussion

In the current strategy of laparoscopic repair of PPU, our results showed that the combination of barbed suture with modified Graham’s patch can provide acceptable outcomes with reduced surgical time compared with the traditional laparoscopic intracorporeal suture technique. There are many scores for evaluating whether MIS or laparotomy is appropriate for the procedure, such as the PULP score, MPI index or Boey score [8]. The Boey score was used at our hospital for its quick and convenient preoperative evaluation to select suitable patients for the laparoscopic approach and patients with Boey’s score ≥ 2 were selected to open surgery at initial diagnosis.

According to the guidelines from the World Journal of Emergency Surgery (WSES), treatments of PPU include early recognition, adequate resuscitation and broad-spectrum antibiotics. Surgical intervention was the first recommended treatment without delay [9, 10]. For huge, perforated ulcers (larger than 2 cm), resection was recommended for lesions at the gastric location and +/- pyloric exclusion for lesions at the duodenal location. For perforated ulcers smaller than 2 cm, primary closure was recommended, and modified Graham’s repair for peptic ulcer perforation was reassessed as an effective procedure to give excellent outcomes in terms of healing, morbidity and mortality [11]. The use of vascularized pedicle omentum can help heal the ulcer and prevent cutting through and ulcer recurrence.

Laparoscopic approach provides significant advantages with less postoperative pain in the first postoperative day and less postoperative surgical site infection with comparable overall postoperative mortality, leak of the suture repair, interloop abscess and reoperation rate if the patients have relatively stable vital signs [12]. Laparoscopic repair for perforated peptic ulcers with omentum patch enforcement has been recommended since 1990 [13], and this procedure was suggested by a randomized controlled study as a first-line option for PPU patients. Even with the evolution of laparoscopic training programs, the complexity of intracorporeal suturing and knot-tying is still the main barrier to advancing minimally invasive surgery.

We presented our simple and effective suture technique in combination with barbed sutures with pedicled omentum patches, which could shorten the total operation time with noninferior outcomes and provide opportunities for beginners to practice. From our data, the operative time was significantly shorter in the barbed suture group than in the traditional suture group, but there was no significant difference in the surgical outcome. The position of patients after anesthesia would be changed to reverse Trendelenburg position during the operation of upper abdomen. During previous operations, using traditional interrupted ties was a straightforward process. However, when we employ the Modified Graham patch technique, where the omentum patch covers the perforation rather than the suture line, certain challenges arise. The omentum would slip down before the initial tie, and occasionally, confusion about the sequencing of suture ties can extend the operation time. As a regional teaching hospital, we acknowledge that our residents may not possess extensive experience with intracorporeal suturing. This inexperience may contribute to longer operation times in the traditional approach.

We adopted our method to barbed suture, the undirected stich of the suture line can anchor the omentum and we can make the loop before closure of the perforation which wound prevent the time wasting in ometum slipping and intracorporeal tying.We therefore do not need an additional port for the instrument to fix the omentum during the intracorporeal knot tie. Primary suturing of the perforation would have the risk of delayed stenosis after healing of the perforation, and the omentum patch not only helps to enforce the repair but also prevents further stenosis. No patient in our series had delayed stenosis during the follow-up period in our series.

The modified GOALS score evaluates psychomotor coordination, including depth perception, bimanual dexterity, efficiency and tissue handling and verbal cues [14] It takes much time to become familiar with complex coordination to perform a safe intracorporeal sut.ure, even for experienced surgeons. Many training programs aim to prescribe simulation systems with visual accuracy to reduce the learning curve of advanced laparoscopic techniques, but in the real world, surgeons still need practice to overcome the gap between training and practice. With the advancement of laparoscopic surgical skills and the evolution of equipment, intracorporeal suturing can be easily performed with unidirectional barbed sutures. By using barbed sutures for primary closure of perforated peptic ulcers, the design could shorten the operation time, lower the threshold of surgical technique requirements and promise a balance between resident training and patient safety.

Although the number of leakages was slightly increased in the V group, we modified our procedure initiated in the apex of the perforation as much as possible, and sometimes we needed three bites of the suture to prevent leakage from the angle. After that, the complications of leakages decreased dramatically. The senior resident can thus be trained to be familiar with the surgical skills applied to the laparoscopic approach even in emergent surgery and not affect the patient’s safety. In the modern era, training and patient safety are equally emphasized, but we need to strike a balance between them. Barbed suture is a good choice of surgical training without influencing the patient’s outcome and should be considered in the training of advanced laparoscopic techniques, even for beginners in laparoscopic surgery.

Our institution is a regional teaching hospital with limited operation room resources and manpower for emergent operations. The shorter operative time can help to make a faster turnover rate of the operation and less delay of the next emergent operation on the waiting list. The decreased timing of the operation can also help to decrease the cost of anesthesia. The patients were sent to our ER without delay, and the hemodynamics were relatively stable for our group to receive laparoscopic operation after adequate resuscitation. With a quicker operation, patients with less cardiopulmonary reservation may have the chance to receive laparoscopic repair. There were 2 mortalities due to suture leaks in our patients: 1 refused further surgical intervention due to the family’s decision, and the other received reoperation but died due to sepsis progression with multiorgan failure. The mortality rate was comparable with a previous report of 7–9% [15, 16]; thus, this procedure is simple and effective for well-selected patients. With the assistance of the omentum, low output leakage without panperitonitis can be managed conservatively without a repeat operation and can achieve secondary healing under adequate nutritional support and infection control.

To the best of our knowledge, this is not the first report comparing laparoscopic barbed suture with conventional suture for perforated peptic ulcers. However, we used a different technique for modified Graham’s patch re-enforcement with a reduced trocar port, as previously described. Additionally, we emphasized that this is a safe and feasible way for less experienced surgeons to be familiar with laparoscopic skills training. The postoperative outcomes were comparable with even much shorter operation times.

There are some limitations in this study. This is a retrospective study with a small sample size and personal skill bias. Further randomized controlled trials are required to validate our results.

## Conclusion

In this study, we propose the laparoscopic repair of perforated peptic ulcers (PPU) using a combination of the modified Graham’s patch technique and barbed sutures. Our aim is to reduce operative time while achieving patient outcomes comparable to those obtained with traditional interrupted sutures. Furthermore, this approach offers beginners an opportunity to enhance their laparoscopic skills without compromising patient safety.

## Data Availability

All data is contained within the manuscript. The datasets used and analyzed. during the current study available from the corresponding author on reasonable. request.
